# The Catalytic Domain of Neuropathy Target Esterase Influences Lipid Droplet Biogenesis and Lipid Metabolism in Human Neuroblastoma Cells

**DOI:** 10.3390/metabo12070637

**Published:** 2022-07-12

**Authors:** Lin He, Feifei Huang, Yu Wang, Yijun Wu, Li Xu, Pingan Chang

**Affiliations:** 1 Chongqing Key Laboratory of Big Data for Bio-Intelligence, School of Bio-Information, Chongqing University of Posts and Telecommunications, Chongqing 400065, China; s190502002@stu.cqupt.edu.cn (L.H.); huangff@cqupt.edu.cn (F.H.); yuwangbio@gmail.com (Y.W.); 2 Laboratory of Molecular Toxicology, State Key Laboratory of Integrated Management of Pest Insects and Rodents, Institute of Zoology, Chinese Academy of Sciences, Beijing 100101, China; wuyj@ioz.ac.cn; 3 State Key Laboratory of Silkworm Genome Biology, College of Sericulture, Textile and Biomass Sciences, Southwest University, Chongqing 400715, China

**Keywords:** lipid droplet, endoplasmic reticulum, lipid metabolite, neuropathy target esterase, catalytic domain

## Abstract

As an endoplasmic reticulum (ER)-anchored phospholipase, neuropathy target esterase (NTE) catalyzes the deacylation of lysophosphatidylcholine (LPC) and phosphatidylcholine (PC). The catalytic domain of NTE (NEST) exhibits comparable activity to NTE and binds to lipid droplets (LD). In the current study, the nucleotide monophosphate (cNMP)-binding domains (CBDs) were firstly demonstrated not to be essential for the ER-targeting of NTE, but to be involved in the normal ER distribution and localization to LD. NEST was associated with LD surface and influenced LD formation in human neuroblastoma cells. Overexpression of NEST enhances triacylglycerol (TG) accumulation upon oleic acid loading. Quantitative targeted lipidomic analysis shows that overexpression of NEST does not alter diacylglycerol levels but reduces free fatty acids content. NEST not only lowered levels of LPC and acyl-LPC, but not PC or alkyl-PC, but also widely altered levels of other lipid metabolites. Qualitative PCR indicates that the increase in levels of TG is due to the expression of *diacylglycerol acyltransferase* 1 gene by NEST overexpression. Thus, NTE may broadly regulate lipid metabolism to play roles in LD biogenesis in cells.

## 1. Introduction

As a highly expressed protein in the nervous system, neuropathy target esterase (NTE) was identified originally in brain tissue homogenates as a primary target of neuropathic organophosphorus (OP) compounds that cause a delayed toxicity characterized by the degeneration of long axons and the paralysis of the lower limbs [1,2]. NTE is now known as patatin-like phospholipase domain-containing protein 6 (PNPLA6) because of the presence of the patatin domain [3,4]. There are two distinct functional regions in human NTE. One is the amino-terminal region (amino acids 1–680) including an N-terminal transmembrane domain (TMD) and a regulatory (R) region with three putative cyclic nucleotide monophosphate (cNMP)-binding domains (CBDs); the other is the carboxyl-terminal catalytic (C) region (residues 681–1327) containing a patatin domain predicted to mediate enzyme activity [5].

NTE is localized to the endoplasmic reticulum (ER) and the N-terminal TMD is sufficient to target NTE to the ER [6,7,8]. Except for the N-terminal TMD that is anchored on the ER membrane, most of the NTE molecule is exposed to the cytoplasmic face of the ER membrane [6,9]. Although the R-region is not associated with the ER membrane, it is required for the normal ER position of NTE due to the aggregation of NTE deleting the R-region [6,8]. In contrast, the C-region is not involved in the ER targeting of NTE, but acquires catalytic competence in the absence of N-terminal region [6]. NTE functions as a (lyso)phospholipase with a preference for lysophosphatidylcholine (LPC) and phosphatidylcholine (PC) [10,11]. Knockout of *Nte* in the mouse brain causes the accumulation of PC [12], while expression of recombinant NTE decreases the levels of LPC in mammalian cells [13]. Similarly, PC and LPC levels are elevated and decreased in *Swiss cheese* (*sws*), the *Drosophila* orthologue of mammalian *NTE*, mutant and additional sws-expressing flies respectively [14,15,16,17]. Three critical active sites, Ser966, Asp960 and Asp1086, are proposed to constitute a catalytic triad [18]. The NTE esterase region (NEST, amino acids 727–1216) exhibits the enzymatic activity of full-length NTE and hydrolyzes several membrane lipids in vitro [19]. 

Systemic inhibition of a sufficient quantity of NTE with certain OP compounds has led to OP compound-induced delayed neurotoxicity (OPIDN) [1,2]. In addition, with high levels in the nervous system, NTE is expressed ubiquitously in non-neural tissues, such as testis, lung, spleen and kidney [2]. Global knockout of the *Nte* gene in mice impairs vasculogenesis and causes lethality during embryogenesis [20,21]. Apart from this, NTE plays critical roles in the nervous system. The conditional loss of brain NTE and knockdown of NTE results in the suppression of neuron axon outgrowth, neurodegeneration and motor neuron defects in mice and zebrafish [7,22]. NTE is also required for axon maintenance and glial ensheathment of Remak’s fibers in adult mice [12,23]. In fruit fly, the *sws* is essential for the structural maintenance and survival of neurons and glia [14,24,25,26]. Progressive neurodegeneration in the brain, decline of locomotor ability and reduction of lifespan were observed in the flies with *sws* mutation or deletion [14,24,27,28].

In recently years, more and more human *NTE*/*PNPLA6* mutations have been identified to be linked to various complex nervous system diseases, such as hereditary spastic paraplegia 39 (SPG39), Boucher–Neuhäuser syndrome, Gordon–Holmes syndrome, Oliver–McFarlane syndrome and Laurence–Moon syndrome [29,30]. The lipid droplet (LD) is the cellular storage organelle for neutral lipids, such as triacylglycerol (TG) and sterol ester (SE). In addition to energy maintenance and membrane lipids homeostasis, the roles of LDs in the nervous system have now received more and more attention [31,32,33]. Several neurodegenerative disorders, including hereditary spastic paraplegia (HSP) and motor neuron disease (MND), have been demonstrated to be related to LDs [34,35,36]. Genetic disruption of *sws* in flies altered contents of TG and LDs. The levels of TG in *sws* mutant and transgenic flies’ brains were decreased and elevated, respectively [14]. Accumulation of LDs was observed in the brain neuron of *sws* knockdown flies [27]. Our previous results showed that knockdown of NTE did not affect TG content; however, stable expression of NEST induced higher TG levels in human neuroblastoma cells [8]. In the current study, the influence of NEST expression on LD biogenesis and lipids metabolism were further explored by quantitative targeted lipidomics technologies.

## 2. Results

### 2.1. Functional Contribution of CBDs to the ER and LD Targeting of NTE

**Figure 1:**
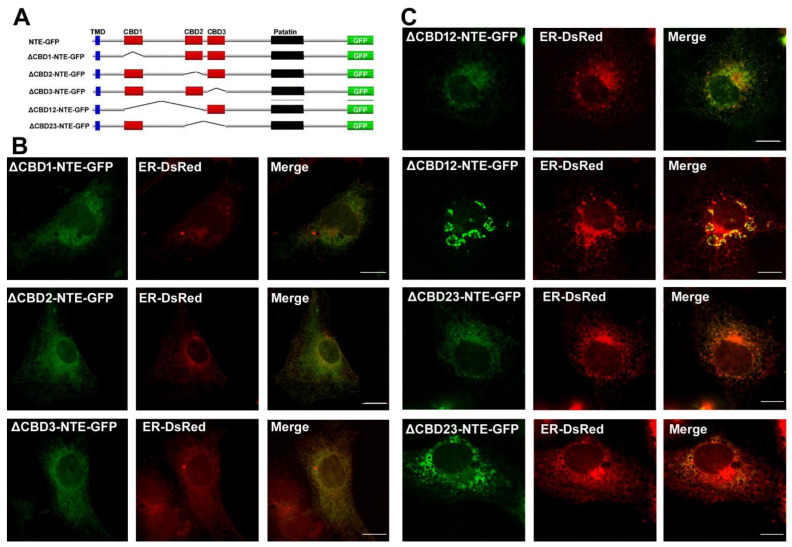
Functional contribution of CBD in NTE to ER targeting and aggregation. (**A**) domain architecture of NTE variants used in this experiment. (**B**,**C**) subcellular distribution of NTE variants with loss of one (**B**) and two CBDs (**C**). COS-7 cells were cotransfected with NTE-GFP truncation mutants and DsRed-ER, a recombinant DsRed2-tagged marker of the ER, and observed by confocal fluorescence microscopy. Figures are representative of at least three experiments. Scale bars = 10 μm.

NTE is anchored on the ER membrane through its N-terminal TMD [6,8]. The R-region is essential for the normal ER distribution of NTE, although it is in the cytoplasm and not associated with the ER membrane [6,8]. However, the role of three CBDs in the R-region of NTE in the ER localization is not well known. Here, various NTE truncated mutants that deleted one CBD alone or two continuous CBDs were constructed to explore the contribution of CBD to ER targeting of NTE (Figure 1A). The correct expression of NTE truncated mutants was detected by immunoblotting (Supplementary Figure S1). As shown in Figure 1B, three NTE truncated mutants losing any one CBD alone displayed a reticular staining pattern, which was completely colocalized with an ER marker, ER-DsRed. The ER was normally distributed without aggregation, which is similar to the ER in NTE moderately expressing cells [8]. When two continuous CBDs were deleted, ∆CBD12-NTE-GFP and ∆CBD23-NTE-GFP with moderate fluorescence still showed a normal ER-pattern subcellular distribution (Figure 1C). However, ∆CBD12-NTE-GFP and ∆CBD23-NTE-GFP that were highly expressed with intense fluorescence localized to the ER with abnormal distribution in the juxtanuclear area, indicating the aggregation of ER (Figure 1C). This is similar to the NTE truncated mutant ∆R-NTE-GFP losing the entire three CBDs (Supplementary Figure S2). Thus, the CBD was not essential for the ER targeting of NTE, but was involved in the normal ER distribution.

**Figure 2:**
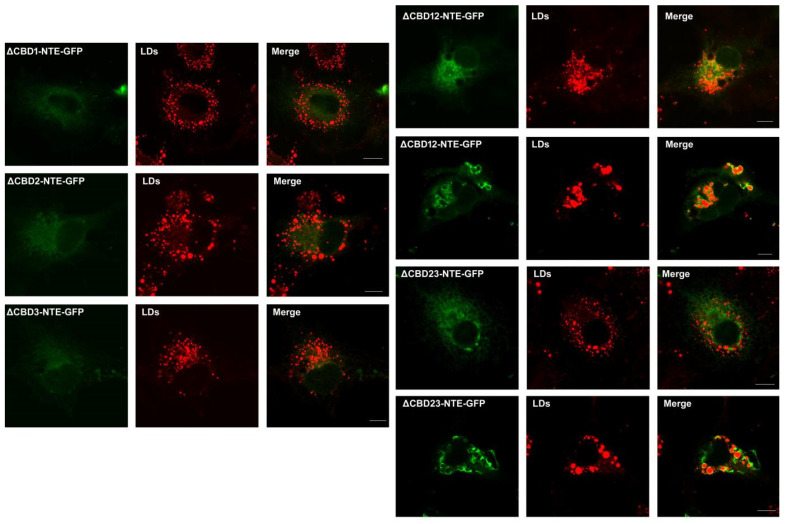
Functional contribution of CBD in NTE to LD targeting. After tranfection with NTE-GFP truncation mutants for 24 h, COS-7 cells were loaded with 0.2 mM for 12 h to induce LD formation. LDs were stained with HSC LipidTOX^TM^ Deep Red neutral lipid stain and images were acquired by confocal fluorescence microscopy. Figures are representative of at least three experiments. Scale bars, 10 μm.

NTE does not attach to the surface of LDs because of the blocking effect of the R-region on the binding of NEST to LDs [8]. It is unknown whether the CBDs in the R-region of NTE contribute to this effect. As shown in Figure 2, when COS-7 was incubated with oleic acid (OA) to enhance TG biosynthesis and LD formation, NTE truncated mutants deleting any one CBD alone did not localize to LDs with a normal ER staining pattern, indicating that lacking each CBD by itself is not sufficient to abolish the inhibitory influence of the R-region on the LD targeting of NEST. ∆CBD12-NTE-GFP and ∆CBD23-NTE-GFP with moderate fluorescence still did not colocalize with LDs, showing a normal ER-pattern subcellular distribution (Figure 2). In contrast, when ∆CBD12-NTE-GFP and ∆CBD23-NTE-GFP were highly expressed, they were aggregated and associated with LDs in an incompletely enclosed manner. Thus, the influence of two continuous CBDs on the LD targeting may be related to their expression levels.

### 2.2. Effect of NEST on LD Biogenesis in Human Neuroblastoma Cells

**Figure 3:**
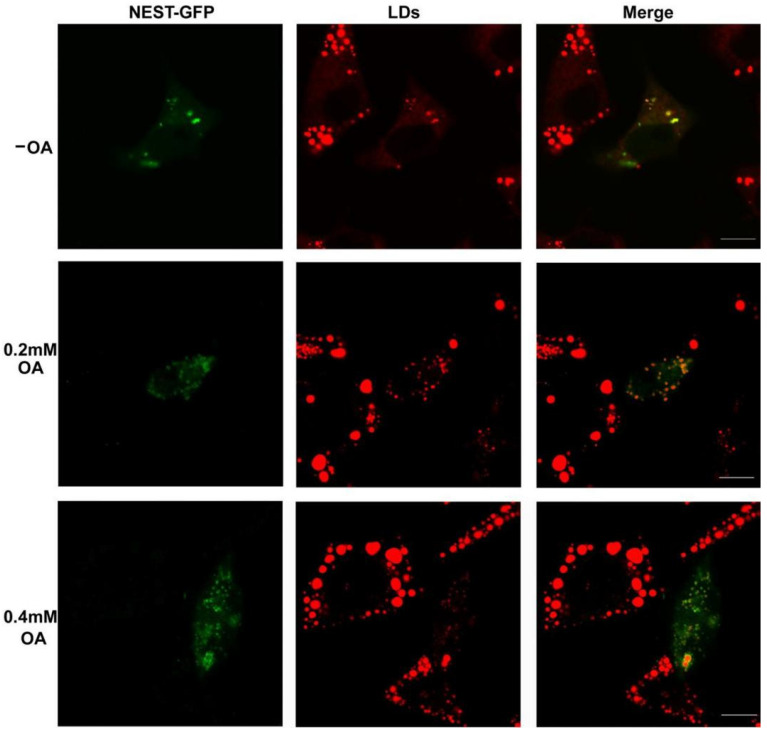
Effect of NEST transient expression on LDs in human neuroblastoma cells. SK-N-SH cells were transfected with NEST-GFP for 24 h, and then incubated with 0.2 mM and 0.4 mM OA for 12 h to induce LD formation or not (−OA). LDs were stained with HSC LipidTOX^TM^ Deep Red neutral lipid stain before image acquisition by confocal fluorescence microscopy. Figures are representative of at least three experiments. Scale bars, 10 μm.

The C-region of NTE (NEST) bound to LDs [8]; however, whether NEST influences LD formation is less clear. As shown in Figure 3, there are a few small LDs in NEST-GFP-expressing human neuroblastoma SK-N-SH cells that were not incubated with OA. In contrast, in the adjacent cells without NEST-GFP expression, more and larger LDs appeared. When cells were incubated with 0.2 mM OA overnight to enhance LDs biogenesis, more small, dispersed LDs were persistent in the cells with expression of NEST-GFP. However, LDs were predominantly fused and became larger in the adjacent cells without the expression of NEST-GFP, and became more obvious in cells incubated with higher (0.4 mM) OA. LDs grew to be extremely large in cells without NEST-GFP, whereas they remained smaller and dispersed in cells expressing NEST-GFP. These results indicated that transient expression of NEST inhibited the fusion and growth of LDs.

### 2.3. Stable Expression of NEST Promoted TG Accumulation in Human Neuroblastoma Cells

**Figure 4:**
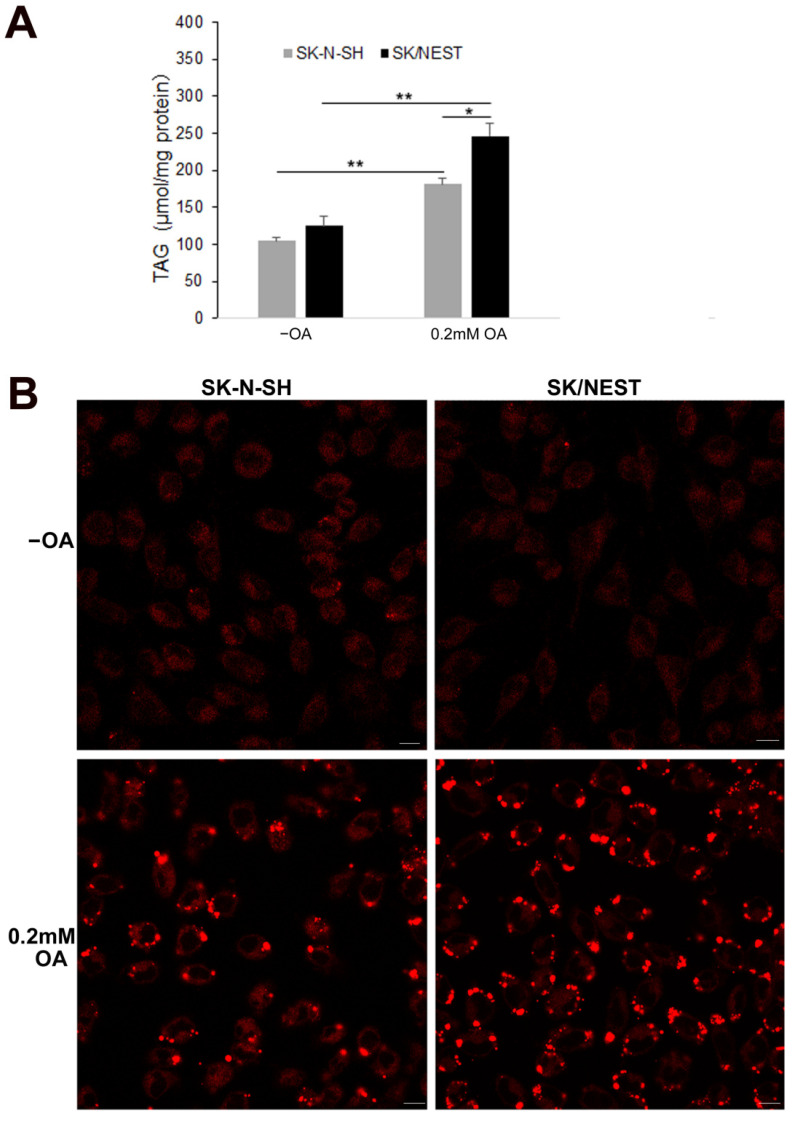
Overexpression of NEST promoted TG accumulation in human neuroblastoma cells. (**A**) TG levels were quantified in control cells (SK-N-SH) and NEST-expressing cells (SK/NEST) in the presence of 0.2 mM OA for 12 h or not (−OA). Data are presented as means ± SD. Asterisks indicate *p* values: * *p* < 0.05, ** *p* < 0.01, *n* = 5. (**B**) LDs were stained with HSC LipidTOXTM Deep Red neutral lipid stain and visualized by confocal fluorescence microscopy. Figures are representative of at least three experiments. Scale bars, 20 μm.

The effect of NEST on contents of TG and LDs formation was further investigated in human neuroblastoma SK/NEST cells stably expressing NEST (Supplementary Figure S3). As shown in Figure 4A, stable overexpression of NEST had no effect on TG levels under normal culture conditions without OA incubation, whereas more TG accumulation was induced in human neuroblastoma cells treated with 0.2 mM OA. This is consistent with previous results at higher concentration of OA [8]. Oil red O staining showed that more LDs with deeper staining were observed in SK/NEST cells than SK-N-SH cells with OA treatment (Supplementary Figure S4). When LipidTOX^TM^ Deep Red was used to image the contents of LDs, OA loading induced large LDs formation in SK-N-SH cells. However, in SK/NEST cells, both large and small LDs appeared to be more abundant than in SK-N-SH cells (Figure 4B). These data suggest that stable expression of NEST promoted accumulation of intracellular TG and LDs.

### 2.4. Overexpression of NEST Altered Levels of TG and FFA

**Figure 5:**
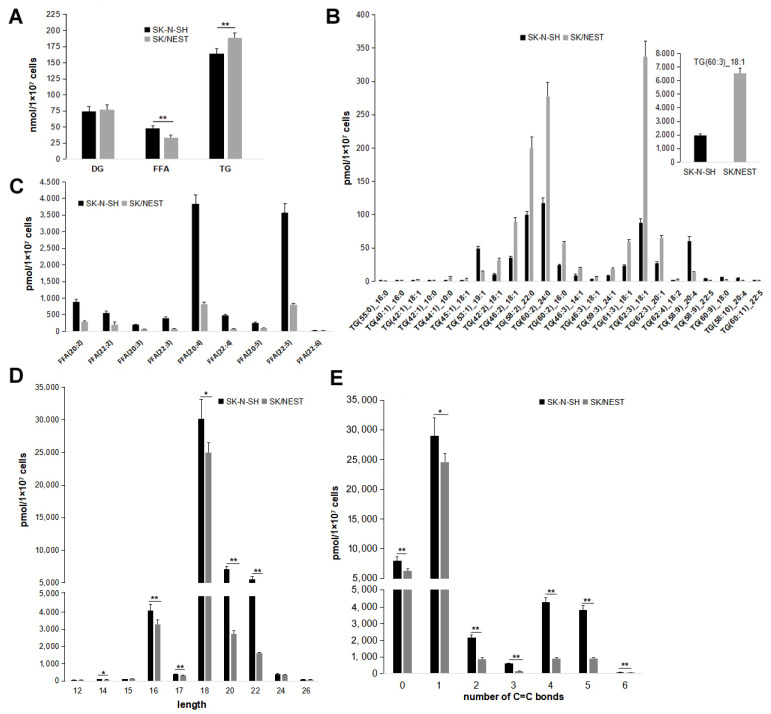
Effect of NEST overexpression on TG, DG and FFA contents in human neuroblastoma cells. SK-N-SH and NEST-overexpressing (SK-NEST) cells were incubated with 0.2 mM OA for 12 h, and then lipids levels were quantified by MetWare based on the AB Sciex QTRAP 6500 LC-MS/MS platform. (**A**) Contents of TG, DG and FFA in SK-N-SH and SK-NEST cells. (**B**,**C**) Comparing the levels of screened TG (**B**) and FFA (**C**) by VIP ≥ 1, FC ≥ 2.0 or FC ≤ 0.50 in SK/NEST and SK-N-SH cells. Data are presented as means ± SD. All *p* < 0.01, *n* = 5. (**D**,**E**) The levels of FFA with different length (**D**) and number of C=C bonds (**E**). Data were presented as means ± SD. Asterisks indicate *p* values: * *p* < 0.05, ** *p* < 0.01, *n* = 5.

Quantitative targeted lipidomic technologies were used to explore the influence of NEST overexpression on intracellular lipids metabolism. A total of 947 lipids were detected through lipidomic analysis, comprising fatty acyl, glycerides, glycerophospholipids, sphingolipids, sterol esters, prenol lipids, glycolipids, etc. TG species were the most abundant, accounting for nearly 30% of total lipids detected (data not shown). Consistent with the previous results, the levels of total TG increased in SK/NEST cells compared with SK-N-SH cells (Figure 5A). However, the content of diacylglycerol (DG), the direct substrate for TG biosynthesis, was not altered, whereas free fatty acid (FFA) levels were significantly reduced by NEST overexpression (Figure 5A).

According to fold change (FC) ≥ 2.0 or FC ≤ 0.50 of lipid metabolites levels between SK-N-SH and SK/NEST cells and variable importance in projection (VIP) ≥ 1, 112 lipid metabolites were screened, of which 87 and 25 lipids were down- and up-regulated respectively in SK/NEST cells (Supplementary Figure S5). A total of 25 TGs were screened out including 18 decreased and 7 increased TG species, most of which contained unsaturated fatty acyl chains and the level of TG (60:3) 18:1 decreased the most (Figure 5B). Overexpression of NEST reduced the content of 9 FFAs to less than 50% of control SK-N-SH cells, all of which were unsaturated fatty acids, with the greatest reduction in arachidonic acid (Figure 5C). Changes in FFA species were further characterized. Levels of most long- and ultra-long-chain FFAs were reduced in SK/NEST cells (Figure 5D). In addition, the content of all FFAs with saturated, monounsaturated and polyunsaturated chains was lower in SK/NEST cells than in control cells (Figure 5E).

### 2.5. Stable Expression of NEST Affected Levels of LPC, but Not PC

**Figure 6:**
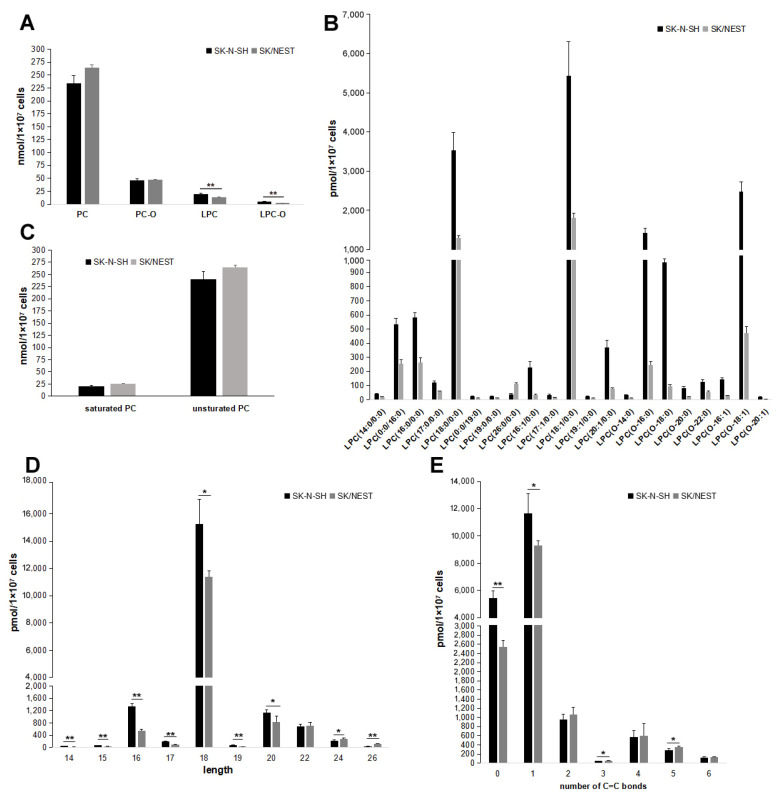
Overexpression of NEST affected the levels of LPC, but not PC in human neuroblastoma cells. (**A**) The levels of acyl-PC (PC), alkyl-PC (PC-O), acyl-LPC (LPC) and alkyl-LPC (LPC-O) were quantified in SK-N-SH and SK-NEST cells after OA incubation for overnight. (**B**) The levels of screened LPC and LPC-O between SK-N-SH and SK/NEST and control according to the standard, VIP ≥ 1, FC ≥ 2.0 or FC ≤ 0.50. Data are presented as means ± SD. All *p* < 0.01, *n* = 5. (**C**) The contents of total PC with saturated and unsaturated fatty acyl groups were compared between SK-N-SH and SK-NEST cells respectively. (**D**,**E**) Comparison of LPC levels with different length of fatty acyls (**D**) and number of C=C bonds (**E**). Data are presented as means ± SD. Asterisks indicate *p* values: * *p* < 0.05, ** *p* < 0.01, *n* = 5.

PC and LPC are the substrates of NTE [10,11]. Ether analogs of PC and LPC, alkyl-PC (PC-O) alkyl-LPC (LPC-O) that carry an ether linked fatty alcohol contrary to conventional acyl chains at the *sn*-1 position were also detected. As shown in Figure 6A, the content of LPC but not PC was lowered in SK/NEST cells, compared with SK-N-SH cells. However, the levels of PC-O and LPC-O were less than those of PC and LPC, respectively, in both SK-N-SH and SK/NEST cells. The content of LPC-O, but not PC-O, was also decreased by NEST overexpression. There were 21 LPC species screened according to FC ≥ 2.0 or FC ≤ 0.50 of lipid metabolites levels, of which 20 LPCs were decreased in NEST-expressing cells. The screened LPCs contained saturated or unsaturated fatty acyl chains and LPC containing oleoyl chain showed the greatest reduction (Figure 6B).

Moreover, no changes in PCs containing saturated or unsaturated acyl chains were observed in SK/NEST cells compared with control SK-N-SH cells (Figure 6C). The levels of LPC species with long fatty acyl chain (14–20 carbon atoms) were lowered in NEST-expressing cells compared with control SK-N-SH cells. In contrast, levels of LPC species with very long fatty acyl chain (more than 20 carbon atoms) were not decreased, or even increased in LPCs with 24 and 26 carbon atoms fatty acyl chain (Figure 6D). The content of LPC species with saturated and mono-unsaturated fatty acyl chain was downregulated by NEST overexpression, whereas the content of LPC species with poly-unsaturated fatty acyl chain was not altered or was upregulated in SK/NEST cells (Figure 6E).

### 2.6. Overexpression of NEST Increased the Expression of DGAT1 and PPARα Genes

**Figure 7:**
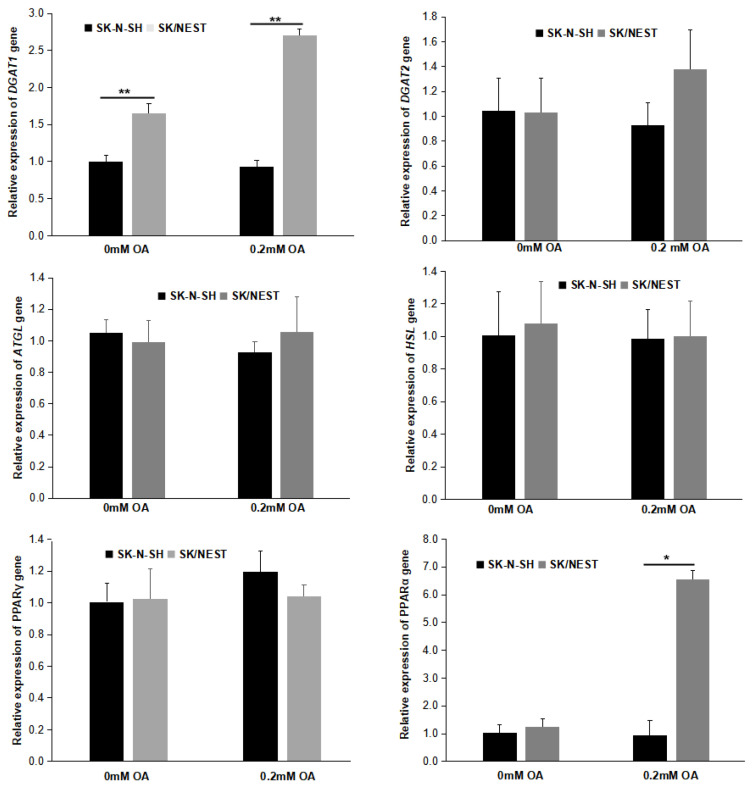
The expression of genes related to TG metabolism affected by NEST overexpression. SK/NEST and SK-N-SH cells were loaded with 0.2 mM OA or not (0 mM OA) for 12 h, and then the total RNA was extracted and transcribed reversely to cDNA. mRNA levels were quantified by qPCR using *β*-*actin* as a reference gene and presented as fold change of SK-N-SH cells without OA treatment. Data are presented as means ± SD. Asterisks indicate *p* values: * *p* < 0.05, ** *p* < 0.01, *n* = 5.

The expression of genes involved in TG metabolism and LD biogenesis was detected by RT-qPCR. As shown in Figure 7, NEST expression increased the mRNA levels of *diacylglycerol acyltransferase 1* (*DGAT1*), but not *DGAT2,* the key enzymes of TG biosynthesis in neuroblastoma cells incubated with or without OA. On the other hand, the expression of TG mobilization genes, *adipose triglyceride lipase* (*ATGL*) and *hormone-sensitive lipase* (*HSL*) was not altered. NEST overexpression did not influence the expression of genes related to fatty acid synthesis, such as *fatty acid synthase* (*FASN*), *acetyl-CoA carboxylase α* (*ACACA*) and *stearoyl-CoA desaturase* (*SCD*) (Supplementary Figure S6). In addition, the expression of *peroxisome proliferator-activated receptor α* (*PPARα*), but not *PPARγ*, was upregulated in SK/NEST cells compared with control SK-N-SH cells after OA loading (Figure 7).

## 3. Discussion

Although the amino-terminal R-region and carboxyl-terminal C region of NTE mainly function in ER-targeting and LD localization respectively [6,8], their roles in subcellular distribution have not been fully understood. Three putative CBDs were persistent in the R-region of NTE; however, no evidence demonstrated that cAMP could bind to CBDs [37]. The R-region was essential for the normal ER position of NTE [6,8]. Here, the role of CBDs in ER- and LD-targeting was further explored. NTE truncated mutants deleting any one CBD alone still localized to the ER and did not associate with LDs or alter the ER morphology. However, NTE truncated mutants losing two continuous CBDs at high levels displayed an abnormal aggregated ER distribution and coincided with LDs at the surface with an unclosed ring pattern. The ER aggregation was thought to result from the intermolecular association of NTEs through their C-region, which was hindered by the R-region to some degree [6]. The R-region also affected the interaction of NTE C-region with LDs [8]. This blocking effect seems to be related to the length of the R-region and the protein expression levels. Normal ER distribution was observed in cells with moderate expression of NTE mutants losing two continuous CBDs. Full length NTE with an entire R-region at high levels also induced abnormal ER accumulation [6,8]. In addition to a role of ER-targeting, CBDs also participated in the function regulation of NTE. NTE mutants with CBD mutations linked to rare complex nervous system syndromes increased the levels of LPC in the fly’s brain [17], although cAMP did not influence the esterase activity of NTE in vitro [6].

LDs with a smaller size were observed in transient NEST-expressing human neuroblastoma cells, compared to the neighbor control cells. The smaller size of LDs may be due to the inhibition of fusion growth of LDs dispersed in the cytoplasm. Our previous findings showed that LDs in NEST-expressing COS-7 cells were comparable in size to control cells and were clustered [8]. This suggests that the effect of NEST on LD formation may be cell-specific. Stable expression of NEST promoted TG accumulation in human neuroblastoma cells incubated with OA. Staining of LDs showed that the number of LDs with large and small sizes was more than those of control cells. In contrast, knockdown of *sws* in the fly brain did not alter the number of LDs of a specific size, although the total LD number of all sizes was elevated [27]. In eukaryotes, de novo formation of LDs originates from the ER and nascent LDs continue to grow via fusion and incorporation of additional neutral lipids. LDs are surrounded by a phospholipid monolayer containing PC, phosphatidylethanolamine (PE), phosphatidylinositol, LPC, and lysophosphatidylethanolamine (LPE), that is decorated with proteins known to regulate LD function [38]. NEST exhibited the phospholipase activity of NTE with preference for LPC and PC [10,11]. NEST was partially associated with the ER in the cytoplasm and translocalized to LDs [6,8]. Thus, the effect of NEST on the biogenesis of LDs may be related to the regulation of phospholipid composition on the surface of LDs.

Quantitative targeted lipidomic analysis showed that the levels of total LPC, but not PC, were reduced in NEST-expressing cells. Neither saturated PCs nor unsaturated PCs levels were altered by NEST overexpression. In contrast, levels of PC were influenced by knockout of *Nte* in the mouse brain and by expression of *sws* mutant in the fly brain [12,14,15,16,17]. In mouse neuroblastoma N2a cells, recombinant NTE downregulated the levels of LPC species with saturated fatty acyls, such as LPC-16:0 and LPC-18:0 [13]. Targeted lipidomic analysis showed that reduction of LPC levels was due to LPC species with long chain, saturated and mono-unsaturated fatty acyl, but not LPCs with very long chain, or poly-unsaturated fatty acyl. The greatest reduction in LPC levels was at LPC-18:1. However, NTE-related esterase, also named PNPLA7, with high homology to NTE, exhibited lysophospholiase activity to control the levels of unsaturated LPC [39]. These data suggest that NTE may directly affect various LPCs, but not PC levels in human neuroblastoma cells. In addition to conventional LPC with a fatty acyl chain at the *sn*-1 position, levels of LPC-O carrying an ether linked fatty alcohol were also reduced by NEST overexpression. As one of the major lipid components of the cell membrane, ether glycerophospholipids (ether-GPLs) constitute a significant component of subcellular membranes, such as the nucleus, ER, post-Golgi network and mitochondria membranes [40]. Ether-GPLs are classified into two types, plasmalogen (1-O-alk-1′-enyl-2-acyl-, i.e., “P”) and alkyl-(1-O-alkyl-2-acyl-, i.e., “O”) GPLs, depending on the substituents at the *sn*-1 position [41]. Ether-GPLs have different physical properties from diacyl-GPLs and have been detected at high levels in brain cells [39,40]. Levels of LPC-O were demonstrated to be associated with the onset and progression of Alzheimer’s disease in mice [42]. LPC-O is not a direct substrate of lysophospholipase, and the mechanism by which NEST affects LPC-O levels remains elusive.

TG levels were elevated by overexpression of NEST in human neuroblastoma cells. This was in line with previous results showing that elevated expression of sws increased TG, while decreased TG levels were observed in the *sws* mutant fly brain [14]. The contents of DG were not altered, but FFA levels declined. The levels of most long and very long chain, saturated, mono- and poly-unsaturated FFAs were lowered by NEST stable expression. Nine long-chain polysaturated FFAs were reduced by more than 2-fold. The accumulation of TG was due to an increased expression of *DGAT1*, but not the *DGAT2* gene. DGAT incorporates fatty acyl into DG to synthesize TG, which is the key enzyme in TG biosynthesis [43]. FFAs liberated from LPC by NEST may be re-routed to TG synthesis. However, the reduction of FFA levels suggested that partial TGs may be synthesized from other FFAs than those liberated from LPC. Alternatively, NEST-induced LPC changes in the LD surface structure may affect TG storage. Another neutral lipid, cholesteryl ester (CE), was also stored in the core of LDs. Levels of total CEs were not altered in NEST-expressing cells (Supplementary Figure S7). The absence of changes in CE levels was detected in NTE knockout mice brains [12]. Thus, NTE could indirectly affect TG, but not CE levels.

In addition, other glycerophospholipids (GPs) metabolisms was indirectly affected (Supplementary Figure S7). The contents of phosphatidylglycerol (PG) were downregulated, while those of phosphatidylinositol (PI) and phosphatidylserine (PS) were increased by NEST overexpression. Meanwhile, the levels of lyso-PG (LPG), lyso-PI (LPI) and lyso-PS (LPS) showed opposite trends to the corresponding GPs. Moreover, sphingolipids metabolism was also altered (Supplementary Figure S7). The levels of ceramide (Cer), phosphoceramide (CerP) and sphingomyelin (SM) were reduced, while those of sphingosine (SPH) were upregulated in SK/NEST cells. These data demonstrated that stable expression of NEST broadly influenced lipid metabolism in human neuroblastoma cells.

Taken together, the effects of CBDs in the NTE R-region on ER position and LD targeting suggested that CBDs affected ER organization and LD targeting via NEST, which correlated with protein expression levels. NEST not only banded to LDs, but also affected LDs biogenesis. More importantly, quantitative targeted lipidomic analysis showed that NEST not only directly catalyzed the deacylation of various LPCs, but not PC, but also widely changed the metabolism of other lipids. The most significantly altered levels of lipid metabolite were TGs, which resulted from the change in *DGAT1* expresssion in human neuroblastoma cells.

## 4. Materials and Methods

### 4.1. Materials

African green monkey kidney fibroblast-like COS-7 cells were purchased from the Cell Center of the Chinese Academy of Medical Sciences (China). Human neuroblastoma SK-N-SH and SK/NEST cells that stably expressed NEST were from our previous study [44]. The NTE-GFP and ΔR-NTE-GFP constructs were generous gifts from Dr. Paul Glynn [6]. Plasmid pEGFP-N3 was purchased from Clontech (Mountain View, CA, USA). Transfection reagent Lipofectamine 2000 and HCS LipidTOX™ Deep Red neutral lipid stain were purchased from Thermo Fisher Scientific (Waltham, MA, USA). Cell culture reagents and OA were from Sigma-Aldrich (St. Louis, MO, USA). The E1003 triglyceride assay kit was purchased from Applygen Technologies (Beijing, China). The Q5 Site-Directed Mutagenesis Kit was purchased from New England Biolabs (Ipswich, MA, USA). Mouse anti-GFP, anti-GAPDH monoclonal antibodies, and goat anti-mouse IgG HRP were from Santa Cruz Biotechnology (Santa Cruz, CA, USA). Enhanced chemiluminescence (ECL) reagents were obtained from Beyotime Biotechnology (Shanghai, China).

### 4.2. Plasmids Construction

NTE CBD truncated mutants were generated using the Q5 Site-Directed Mutagenesis Kit with NTE-GFP as the template and primers indicated in Table 1. All plasmids were sequenced to confirm the presence of the desired mutations.

### 4.3. Cell Culture and Transfection

COS-7 and SK-N-SH cells were cultured in Dulbecco’s modified Eagle’s medium (DMEM) supplemented with 10% fetal bovine serum, 100 units/mL penicillin and 100 μg/mL streptomycin in a 37 °C incubator with 5% CO_2_. SK/NEST cells overexpressing NEST were maintained in DMEM containing 200 μg/mL G418 [44]. Cell transfection was performed using Lipofectamine 2000 transfection reagent. COS-7 cells were co-transfected with GFP, NTE-GFP or its mutants and DsRed-ER, the ER marker, for 48 h to observe the colocalization of proteins with the ER. DsRed-ER consists of an ER targeting sequence of calreticulin fused to the N terminus of DsRed and a C-terminal ER retention sequence (KDEL). SK-N-SH cells were transfected with NEST-GFP for 24 h and then loaded with 0.2 mM OA complexed to fat-free BSA for 12 h to induce TG synthesis and LD formation.

### 4.4. Confocal Fluorescence Microscopy

For detection of protein and ER colocalization, COS-7 cells were seeded in a 12-well plate mounted onto cover slips and transfected as described above. After transfection for 48 h, the cells were washed three times with 1× PBS and then fixed with 4% paraformaldehyde (PFA) for 30 min at room temperature. For detection of LDs, COS-7 and SK-N-SH cells that were transfected with NEST-GFP for 24 h, SK-N-SH and SK/NEST were incubated with 0.2 mM and 0.4 mM OA for 12 h, and then fixed. LDs were stained with HCS LipidTOX Deep Red (1:1000 in PBS) for 30 min. Fluorescent images were acquired by confocal scanning microscopy with a Leica SP5 confocal microscope equipped with a Leica HCX 63 × 1.4 NA oil immersion objective. GFP was excited at 488 nm, and emission was detected between 500 and 530 nm. DsRed was excited at 561 nm, and emission was detected between 580 and 610 nm. HCS Lipid^TOX^ Deep Red was excited at 633 nm, and emission was detected between 650 and 700 nm. All the presented experiments were repeated independently at least 3 times.

### 4.5. Measurement of TG Levels

Briefly, cells were washed with PBS twice and harvested, and then dissolved in lysis buffer. The TG content and protein concentration of the supernatant were determined by an enzymatic triglyceride assay kit (Applygen, Beijing, China) and a BCA protein assay kit (Beyotime Biotechnology, Shanghai, China).

### 4.6. Quantitative Targeted Lipidomic Analysis

Human neuroblastoma SK-N-SH and SK/NEST cells were incubated with 0.2 mM OA for 12 h and then every 1 × 10^7^ cells were collected in one tube. Each group set 5 repeats. Samples were placed in liquid nitrogen for 2 min, then thawed on ice for 5 min and vortexed to mix. After freezing and thawing 3 times, samples were then centrifuged with 5000 rpm at 4 °C for 1 min. Lipids were extracted with 1 mL mixture containing the following methyl tert-butyl ether: methanol = 3:1 and internal standard mixture. After adding 200 μL water, the sample was centrifuged with 12,000 rpm at 4 °C for 10 min. The supernatant (500 μL) was extracted and concentrated, and then dissolved in 200 μL mobile phase B for LC-MS/MS analysis.

The sample extracts were analyzed using an LC-ESI-MS/MS system (UPLC, ExionLC^TM^ AD, https://sciex.com.cn/; MS, QTRAP^®^ 6500+ System, https://sciex.com.cn/). The analytical conditions were as follows, UPLC: column, Thermo Accucore™ C30 (2.6 μm, 2.1 mm × 100 mm i.d.); solvent system, A: acetonitrile/water (60/40, *v*/*v*, 0.1% formic acid, 10 mmol/L ammonium formate), B: acetonitrile/isopropanol (10/90, *v*/*v*, 0.1% formic acid, 10 mmol/L ammonium formate); gradient program, A/B (80:20, *v*/*v*) at 0 min, 70:30 *v*/*v* at 2.0 min, 40:60 *v*/*v* at 4 min, 15:85 *v*/*v* at 9 min, 10:90 *v*/*v* at 14 min, 5:95 *v*/*v* at 15.5 min, 5:95 *v*/*v* at 17.3 min, 80:20 *v*/*v* at 17.3 min, 80:20 *v*/*v* at 20 min; flow rate, 0.35 mL/min; temperature, 4 °C; injection volume: 2 μL. The effluent was alternatively connected to an Electrospray Ionization (ESI)-triple quadrupole-linear ion trap (QTRAP)-MS.

Linear ion trap (LIT) and triple quadrupole (QQQ) scans were acquired on a triple quadrupole-linear ion trap mass spectrometer (QTRAP), QTRAP^®^ 6500+ LC-MS/MS System, equipped with an ESI Turbo Ion-Spray interface, operating in positive and negative ion mode and controlled by Analyst 1.6.3 software (Sciex). The ESI source operation parameters were as follows: ion source, turbo spray; source temperature 500 °C; ion spray voltage (IS) 5500 V (Positive), −4500 V (Negative); Ion source gas 1 (GS1), gas 2 (GS2), curtain gas (CUR) were set at 45, 55, and 35 psi, respectively. Instrument tuning and mass calibration were performed with 10 and 100 μmol/L polypropylene glycol solutions in QQQ and LIT modes, respectively. QQQ scans were acquired as MRM experiments with collision gas (nitrogen) set to 5 psi. Decluttering potential (DP) and collision energy (CE) for individual multiple reaction monitoring (MRM) transitions was done with further DP and CE optimization. A specific set of MRM transitions were monitored for each period according to the metabolites eluted.

The MWDB (metware database, http://www.metware.cn/, accessed on 15 June 2021) was constructed based on the standard materials to qualitatively analyze the data detected by mass spectrometry [45]. Lipids contents were detected by the MWDB based on the AB Sciex QTRAP 6500 LC-MS/MS platform. The fold change (FC) of the nonparametric test and variable importance in projection (VIP) calculated on the basis of the orthogonal partial least squares discriminant analysis (OPLS-DA), were used in combination to screen the differential lipid metabolites. Then, differential lipid metabolites between SK-N-SH and SK/NEST cells were screened according to the standard cut offs of VIP ≥ 1, FC ≥ 2.0 or FC ≤ 0.50.

### 4.7. RNA Extraction and RT-qPCR

RNA was prepared using an EASYspin plus RNA extraction kit (Aidlab technologies, Beijing, China) and then transcribed to cDNA with an ueIris RT mix with DNase (All-in-One) kit (Yuheng Biotechnologies, Suzhou, China) according to the manufacturer’s instructions. Real-time quantitative PCR (qPCR) was performed with ChamQ SYBR qPCR Master Mix (Vazyme biotechnologies, Nanjing, China) on a QuantStudio 3 Real-Time PCR System. The primers used are shown inTable 2. To account for differences in cell numbers, all cycle threshold (Ct) values of sample replicates were normalized to those of *actin* as the reference gene. Relative mRNA levels were quantified with the ΔΔCt method [46].

### 4.8. Statistical Analysis

Data were generally expressed as mean ± standard deviation (SD) values. Groups of data were compared by one-way ANOVA and by post hoc analysis using the Student–Keuls method. A difference between means was considered significant at *p* < 0.05.

## Figures and Tables

**Table 1:** Primers used to generate NTE truncated constructs.

Construct Name	Primer Name	Primer Sequence (5′ to 3′)
ΔCBD1-NTE-GFP	ΔCBD1F	CCGGAGAGCTTGGTGCGG
	ΔCBD1R	GACCATGTGGCGGCAGAG
ΔCBD2-NTE-GFP	ΔCBD2F	CAGATGGACTTCGCCATC
	ΔCBD2R	CAAGACTCTGCTGTTCAG
ΔCBD3-NTE-GFP	ΔCBD3F	CCGCAGGTCGTGACCCGC
	ΔCBD3R	CCAGTCGATGGCGAAGTCCATCTG
ΔCBD12-NTE-GFP	ΔCBD12F	CAGATGGACTTCGCCATCG
	ΔCBD1R	GACCATGTGGCGGCAGAG
ΔCBD23-NTE-GFP	ΔCBD3F	CCGCAGGTCGTGACCCGC
	ΔCBD23R	CAAGACTCTGCTGTTCAGGAGGGAG

**Table 2:** Primers used in qPCR experiment.

Primer Name	Primer Sequence (5′ to 3′)
hATGLF	GAGATGTGCAAGCAGGGATAC
hATGLR	CTGCGAGTAATCCTCCGCT
hHSLF	GACCCCTGCACAACATGATG
hHSLR	TGAGCAGCACCCTTTGGATG
hDGAT1F	GGTCCCCAATCACCTCATCTG
hDGAT1R	TGCACAGGGATGTTCCAGTTC
hDGAT2F	ATTGCTGGCTCATCGCTGT
hDGAT2R	GGGAAAGTAGTCTCGAAAGTAGC
hFASNF	AAGGACCTGTCTAGGTTTGATGC
hFASNR	TGGCTTCATAGGTGACTTCCA
hSCDF	TTCCTACCTGCAAGTTCTACACC
hSCDR	CCGAGCTTTGTAAGAGCGGT
hACACAF	TCACACCTGAAGACCTTAAAGCC
hACACAR	AGCCCACACTGCTTGTACTG
hPPARαF	TTCGCAATCCATCGGCGAG
hPPARαR	CCACAGGATAAGTCACCGAGG
hPPARγF	TACTGTCGGTTTCAGAAATGCC
hPPARγR	GTCAGCGGACTCTGGATTCAG
hNTEF	CGGGTGCAGAAAACTCCAG
hNTER	CGCATAATCTTCCGGCCATAGA
hactinF	CATGTACGTTGCTATCCAGGC
hactinR	CTCCTTAATGTCACGCACGAT

## Data Availability

Not applicable.

## Supplementary Materials

The following supporting information can be downloaded at: https://www.mdpi.com/article/10.3390/metabo12070637/s1, Figure S1: The expressions of GFP, NTE and its truncation mutants in whole cell lysis were detected by immunoblotting using anti-GFP antibody; Figure S2: NTE deleting the R-region induced ER aggregation. A, domain architecture of ∆R-NTE-GFP. B, ∆R-NTE-GFP was expressed in COS-7 and the ER was marked by co-expression of ER-DsRed. Images were acquired by confocal fluorescence microscopy. Scale bars = 10μm; Figure S3: The expression of NTE gene was quantified by RT-qPCR. mRNA levels in SK-N-SH and SK/NEST cells were quantified by qPCR using actin as a reference gene and presented as fold change of SK-N-SH cells. Data are presented as means ± SD. Asterisk indicated *p* values: * *p* < 0.05, n = 5; Figure S4: Oil red O staining in SK-N-SH and SK/NEST cells. Cells were loaded with 0.2 mM OA for 12 h or not and then stained with oil red O. Figures were representative of three separate experiments; Figure S5: Volcano map showed the deferential metabolites between SK/NEST and SK-N-SH cells selected by VIP ≥ 1, FC ≥ 2.0 or FC ≤ 0.50; Figure S6: The expression of genes related to FA biosynthesis affected by NEST overexpression. mRNA levels were quantified by qPCR using β-actin as a reference gene and presented as fold change of SK-N-SH cells without OA treatment. Data are presented as means ± SD. n = 5; Figure S7: Effect of NEST overexpression on different lipids contents in human neuroblastoma cells. Data were presented as means ± SD. Asterisk indicated *p* values: * *p* < 0.05, ** *p* < 0.01, n = 5.
