# Supplementary material for: The Catalytic Domain of Neuropathy Target Esterase Influences Lipid Droplet Biogenesis and Lipid Metabolism in Human Neuroblastoma Cells
Source: Metabolites. 2022 Jul 12;12(7):637. doi: 10.3390/metabo12070637 (PMC9319352; doi:10.3390/metabo12070637)
Supplement: Supplementary file 1 [file metabolites-12-00637-s001.zip › metabolites-1769754-supplementary.pdf]

Supplementary material

Supplementary methods

Immunoblot analysis

After transfection for 48 h, total protein samples were prepared by sonication in RIPA Buffer containing 1 mM phenylmethanesulfonyl fluoride (PMSF), and then centrifuged at 4°C and 15,000 g for 15 min to pellet the cell debris. The supernatant protein concentration was determined with BCA Protein Assay Kit (Beyotime Biotechnology). The supernatant was mixed with 5 × SDS loading buffer and boiled for 5 min. All the samples were subjected to SDS/PAGE, transferred to nitrocellulose filters and subjected to immunoblotting analysis using anti-GFP antibody (1:1000 dilution) as described previously [42].

Oil red O staining

SK-N-SH and SK/NEST cells were incubated with 0.2 mM OA for 12h. After washing with PBS, cells were fixed with 4% paraformaldehyde in PBS for 10 min at room temperature and then subjected to Oil red O staining. Cell images were acquired using an Olympus IX71 inverted microscope.

Supplementary Figures

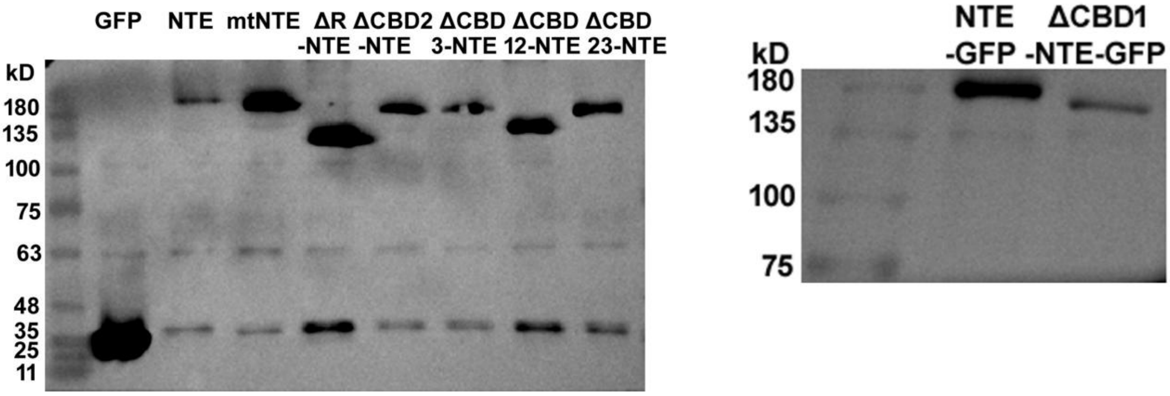

Figure S1. The expressions of GFP, NTE and its truncation mutants in whole cell lysis were detected by immunoblotting using anti-GFP antibody.

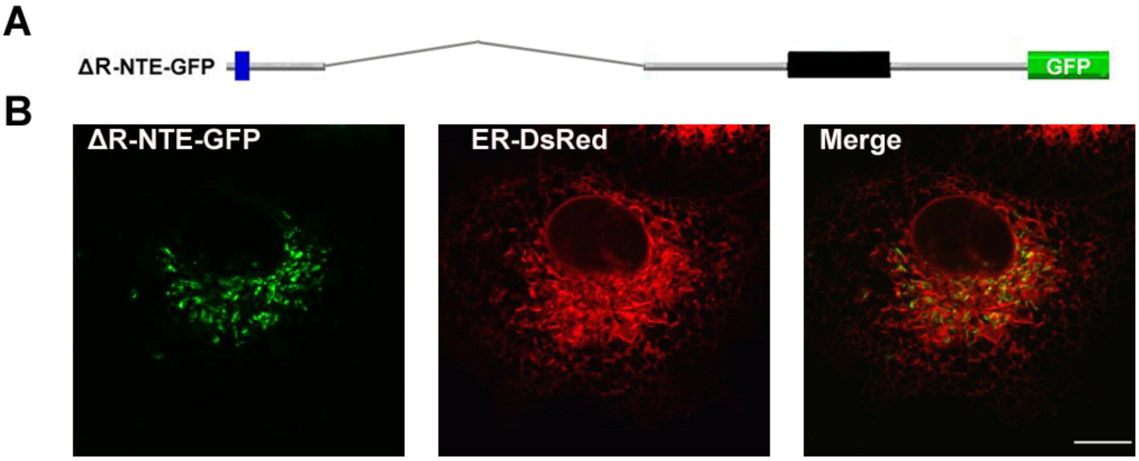

Figure S2. NTE deleting the R-region induced ER aggregation. A, domain architecture of ΔR-NTE-GFP. B, ΔR-NTE-GFP was expressed in COS-7 and the ER was marked by co-expression of ER-DsRed. Images were acquired by confocal fluorescence microscopy. Scale bars = 10μm.

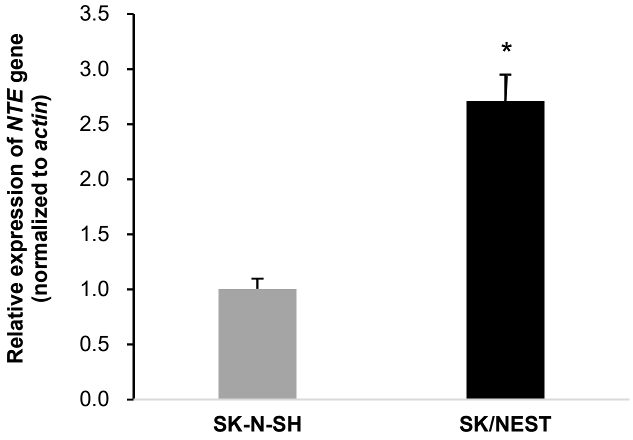

Figure S3. The expression of *NTE* gene was quantified by RT-qPCR. mRNA levels in SK-N-SH and SK/NEST cells were quantified by qPCR using *actin* as a reference gene and presented as fold change of SK-N-SH cells. Data are presented as means ± SD. Asterisk indicated *P* values:* *P* < 0.05, n = 5.

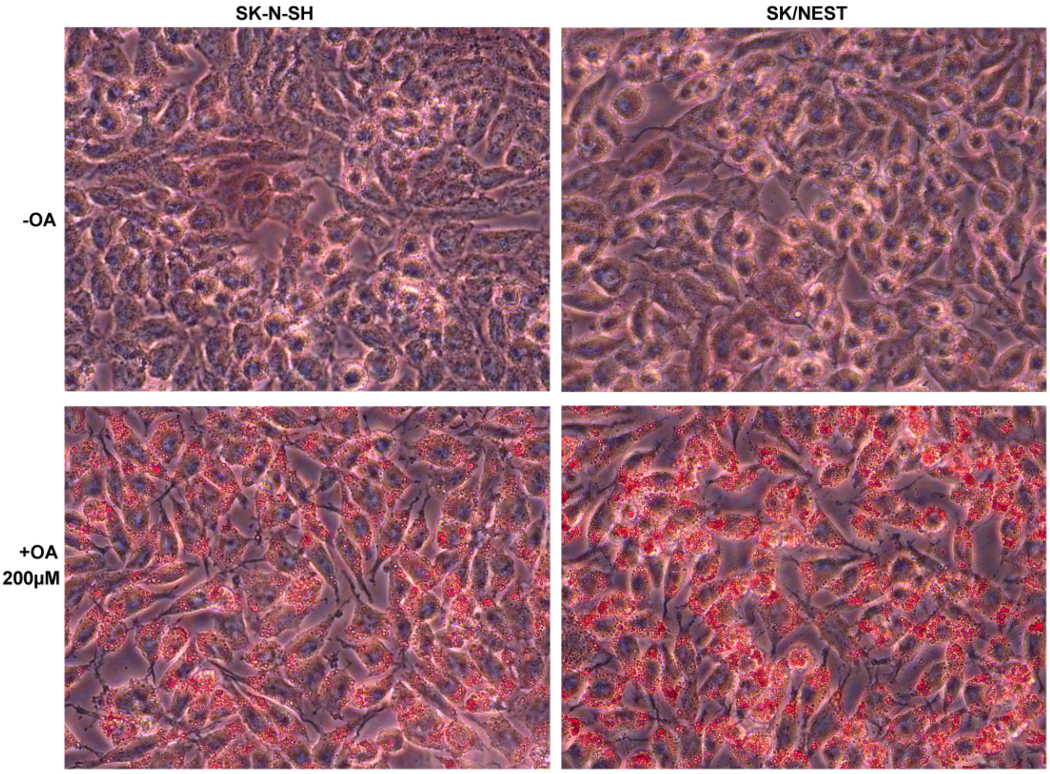

Figure S4. Oil red O staining in SK-N-SH and SK/NEST cells. Cells were loaded with 0.2 mM OA for 12 h or not and then stained with oil red O. Figures were representative of three separate experiments.

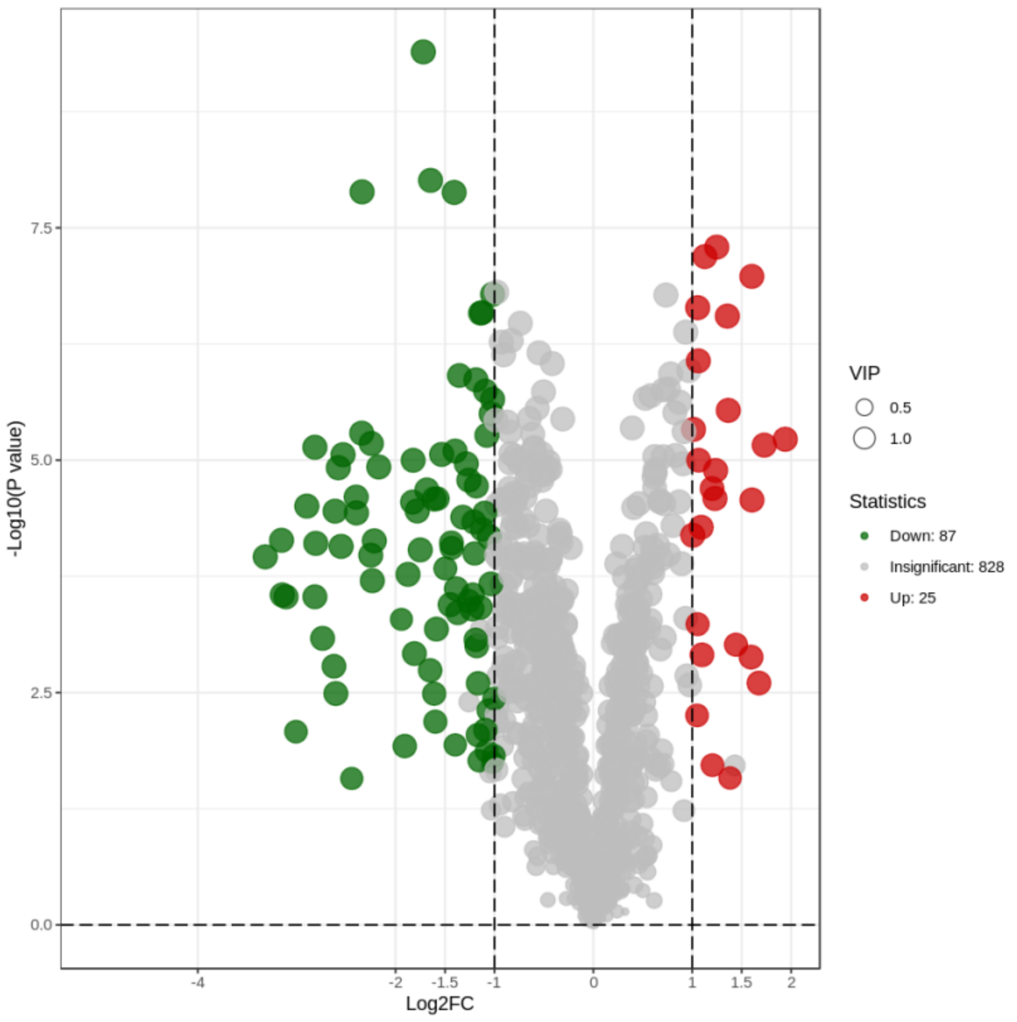

Figure S5. Volcano map showed the deferential metabolites between SK/NEST and SK-N-SH cells selected by VIP ≥ 1, FC≥ 2.0 or FC ≤ 0.50.

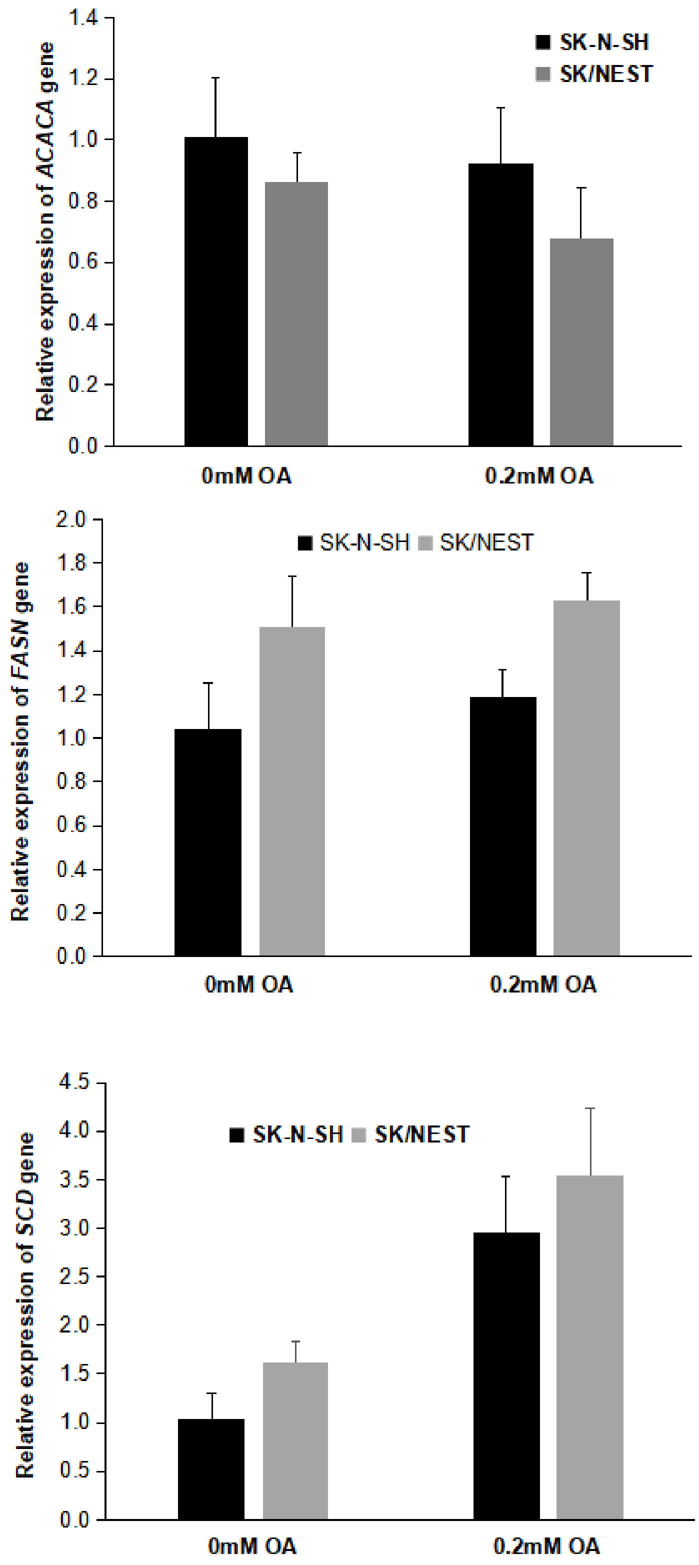

Figure S6. The expression of genes related to FA biosynthesis affected by NEST overexpression. mRNA levels were quantified by qPCR using *β-actin* as a reference gene and presented as fold change of SK-N-SH cells without OA treatment. Data are presented as means ± SD. n = 5.

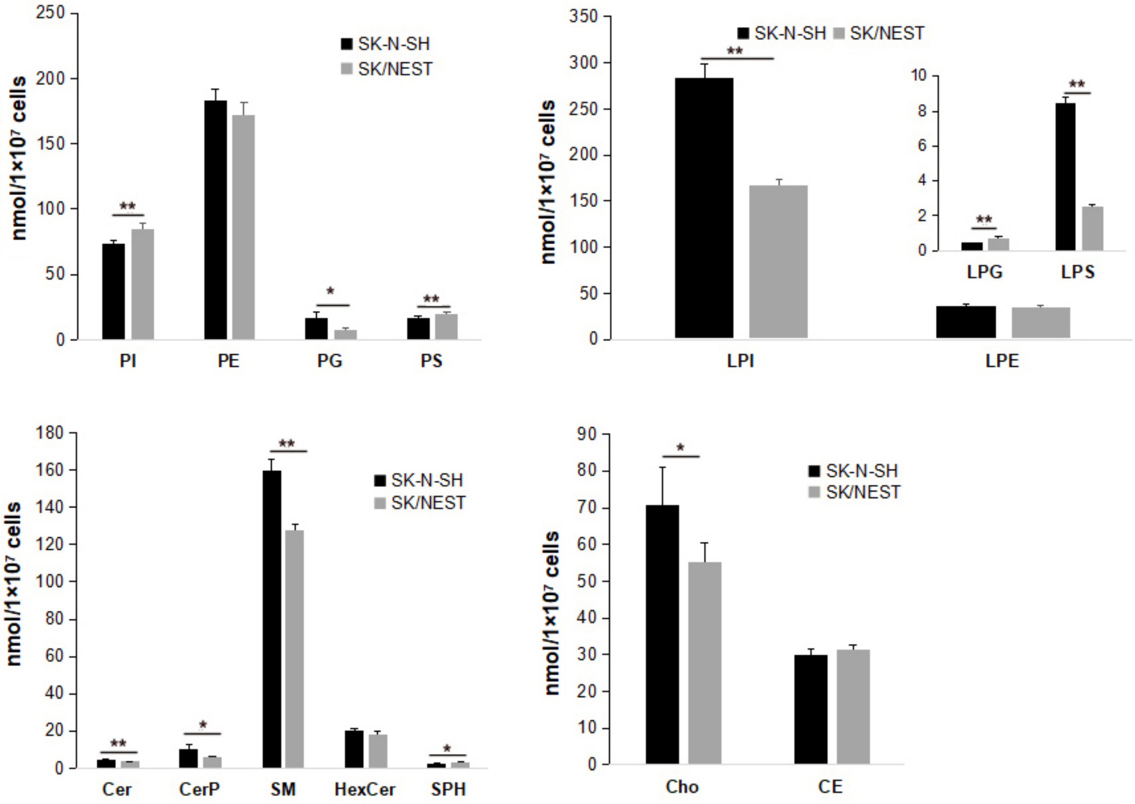

Figure S7. Effect of NEST overexpression on different lipids contents in human neuroblastoma cells. Data were presented as means ± SD. Asterisk indicated p values: * $P < 0.05$, ** $P < 0.01$, n = 5.
